# Pulmonary artery aneurysm and dissection caused by patent ductus arteriosus

**DOI:** 10.1093/eurheartj/ehac438

**Published:** 2022-08-09

**Authors:** Huawei Zhang, Chengxin Weng, Ding Yuan, Tiehao Wang

**Affiliations:** Department of Radiology, West China Hospital, Sichuan University, 37 Guo Xue Alley, Chengdu 610041, Sichuan Province, China; Department of Vascular Surgery, West China Hospital, Sichuan University, 37 Guo Xue Alley, Chengdu 610041, Sichuan Province, China; Department of Vascular Surgery, West China Hospital, Sichuan University, 37 Guo Xue Alley, Chengdu 610041, Sichuan Province, China; Department of Vascular Surgery, West China Hospital, Sichuan University, 37 Guo Xue Alley, Chengdu 610041, Sichuan Province, China

**Figure:**
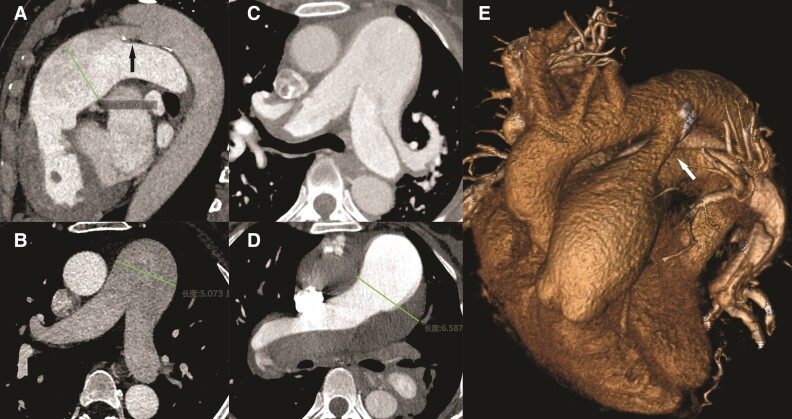


A 52-year-old female was referred to our hospital due to severe chest pain, dyspnoea, and cyanosis. Four years before, she was found to have a patent ductus arteriosus (PDA; arrow, *Panel A*) on contrast-enhanced computed tomography, accompanied by dilation of the pulmonary trunk (5 cm; *Panel B*). No other cardiovascular diseases were discovered. With medical management, her heart condition remained in NYHA functional Class II, and she had full-time employment. The patient, despite the advice of several cardiologists, refused further surgical correction of PDA. On this admission, blood pressure was 90/55 mmHg, and oxygen saturation was 85%. A continuous diastolic and systolic murmur could be heard in the second left intercostal space.

Computed tomographic angiography revealed bilateral pulmonary artery (PA) dissection (*Panel C*) and pulmonary trunk aneurysm (6.5 cm; *Panel D*), associated with a PDA (white arrow, *Panel E*). Echocardiography showed increased PA pressure (97 mmHg) with bidirectional shunt flow. The Eisenmenger’s syndrome (NYHA functional Class IV) was diagnosed, and further Heart–lung transplantation was prepared. PA aneurysm and dissection are very rare complications of pulmonary artery hypertension (PAH), mostly diagnosed at post-mortem examination. Severe PAH caused by PDA usually has a long clinical course, and the accumulation of PAH increases the risk of aneurysm and dissection development. This case illustrated that PDA needs aggressive treatment to prevent PA aneurysm and dissection.

This work was granted by the Sichuan Foundation of Science and Technology (grant number: 2022YFS0359).

The data underlying this article will be shared on reasonable request to the corresponding author.

